# Two cases of pen ink scleral tattoos and a brief review of the literature

**DOI:** 10.1016/j.ajoc.2021.101015

**Published:** 2021-01-20

**Authors:** Austin Rohl, Karen L. Christopher, Cristos Ifantides

**Affiliations:** a Department of Ophthalmology, Sue Anschutz-Rodgers Eye Center, University of Colorado School of Medicine, Aurora, CO, USA; b Rocky Mountain Regional Veterans Affairs Medical Center, Aurora, CO, USA; c Department of Surgery, Denver Health Medical Center, Denver, CO, USA

**Keywords:** Scleral tattooing, Eyeball tattoo, Extreme body modification

## Abstract

**Purpose:**

Scleral tattooing, also known as episcleral, subconjunctival, or simply eyeball tattooing, is a relatively new form of extreme body modification that first emerged in 2007. There are few reports of the management of these tattoos in the medical literature, and we aim to increase the body of knowledge surrounding this rare and potentially dangerous practice.

**Observations:**

We present two new cases of improvised scleral tattooing, both performed in prison using pen ink and insulin needles, and both with minimal complications and managed with topical medications. A brief review of the literature is included which details the dangers of scleral tattooing.

**Conclusions and importance:**

We discuss management of complications for this new, previously unreported method of scleral tattooing using pen ink. Ophthalmologists should be aware of the presentation, possible complications, and management of these cases.

## 1 Introduction

Tattooing of the eye dates to the early 2nd century Roman Empire and was first performed on the cornea. In 150 C E., Galen of Pergamon, an ancient physician and philosopher, first described cauterizing the cornea of patients with unsightly corneal scarring, and then using copper sulfate to dye the area in order to improve cosmesis.^1^ Corneal tattooing is still performed today as a cosmetic treatment for corneal opacities, as well as to treat symptomatic glare from iris defects such as polycoria, traumatic iridodialysis, and laser peripheral iridotomies.^2^, ^3^, ^4^ Corneal tattooing to change perceived iris color has also been described in the medical literature, though this practice remains controversial.^5^^,^^6^

Scleral tattooing, also referred to as episcleral, subconjunctival, or eyeball tattooing, is a more recent form of eye tattooing that falls under the realm of extreme body modification, with the first reports of this practice coming in 2007.^7^ Tattoo ink is injected using fine gauge needles under the conjunctiva into the episcleral tissues. In contrast to corneal tattooing, scleral tattooing is usually performed by non-medical persons, most often tattoo artists or body modifiers. We could find only one example of a scleral tattoo performed by a medical professional, which was done on an anophthalmic socket for cosmetic reasons.^8^

Complications from scleral tattooing are only recently emerging in the medical literature. These range from the less serious surface irritation and conjunctivitis to the more serious uveitis, glaucoma, and endophthalmitis requiring enucleation.^9^, ^10^, ^11^, ^12^, ^13^, ^14^, ^15^, ^16^, ^17^, ^18^, ^19^ A literature review summary of all the previously reported cases of scleral tattooing and their complications are presented in Table 1. Although this is a relatively new procedure, the dangerous nature of scleral tattooing has already been recognized and specific legislation preventing the procedure is now in place in at least four states including Oklahoma, Nebraska, Indiana, and Georgia (Okla. Stat. Title § 21–842.1 (2014), NE LB449 (2019), IN Code § 25-1-19-1 (2018), GA Code § 16-12-5 (2010)).

**Table 1:** Comprehensive list of all previously reported cases of scleral tattooing in the medical literature and their characteristics.

Author	# of cases	Age	Gender	Country	Color Ink	Type of Ink	Complications	Required surgery?	Loss of eye?	Final visual acuity	Other Comments
Chan et al.^9^	3	39	F	Canada	Blue	Tattoo Ink (Starbrite)	Inadvertent puncture wound without injection of ink	Yes - globe exploration	No	Unknown	Recovered well, lost to follow up after 1 week.
		41	M	Canada	Blue	Tattoo Ink (Starbrite)	Inadvertent puncture and injection of 1 cc of ink into anterior chamber	Yes - Anterior chamber washout, tap and inject, pars plana vitrectomy, then pars plana lensectomy and insertion of sulcus lens.	No	20/200	Suspected endophthalmitis but tap negative. Lens zonules dissolved leading to lens subluxation.
		24	M	Canada	Black	Tattoo Ink (metal-free)	Inadvertent puncture and injection of ink	Yes - anterior chamber washout, pars plana vitrectomy, and lensectomy. Enucleation 2 months later.	Yes- *Alcaligenes faecalis* endophthalmitis and complications eventually leading to phthisis requiring enucleation.	Not applicable - Enucleated	Pathology demonstrated staining of the inner retina, sclera, and the corneal endothelium, as well as endothelial cell loss and corneal edema.
Duarte et al.^10^	2	26	M	Mexico	Green	Unknown	Orbital cellulitis and posterior scleritis with choroidal detachment and macular folds.	Yes - Right tarsorrhaphy due to conjunctival exposure.	No	20/25	Subconjunctival penicillin injected during tattooing, and the patient had a known penicillin allergy.
		17	M	Mexico	Orange	Unknown	Episcleral nodules	No	No	20/20	Patient was without pain or decrease in vision.
Cruz et al.^11^	1	25	F	Brazil	Black	Tattoo ink (“Eternal Ink”)	Inadvertent puncture and injection of ink into anterior chamber resulting in severe inflammation, capsular lens opacity, and secondary glaucoma.	Yes - Anterior chamber washout, then trabeculectomy for pressure control.	No	20/100	Had persistent elevations of IOP even after initial washout and required trabeculectomy.
Cruz et al.^12^	1	28	M	Brazil	Blue	Unknown	Conjunctivitis and anterior uveitis	No	No	20/25	Managed with topical drops only
Oswaldo Rodriguez-Avila et al.^13^	1	32	M	Mexico	Red	Tattoo ink	Inadvertent globe penetration	Yes - pars plana vitrectomy	no	20/80	Scanning electron microscopy X-ray microanalysis of the tattoo red ink revealed significant signals of iron, barium, and copper.
Ng et al.^14^	1	34	M	UK	White	Fibracolor Finger Paint	Inadvertent globe puncture	Yes - open globe repair, lensectomy, then subsequent inferior scleral melt requiring sclero-corneal patch graft, amniotic membrane grafting, eventually penetrating keratoplasty.	No	20/125	Was attempting to cover up undesirable scleromalacia from previous eye surgeries. Self-guided technique via YouTube.
Paulo et al.^15^	1	29	M	Colombia	Green	Unknown	Inadvertent globe puncture in both eyes	Yes - anterior chamber washout of both eyes.	No	20/25	Patient was a tattoo artist and had a history of schizophrenia.
Brodie et al.^16^	1	43	M	UK	Red	Tattoo ink	Episcleral nodules	No	No	20/20	First case reported in medical literature. Patient asymptomatic.
Dixon et al.^17^	1	39	M	USA	Green	Tattoo ink	Inadvertent globe puncture in right eye	Yes - globe exploration and pars plana vitrectomy with barrier laser and silicone oil.	No	20/20	Inferior retinal break with localized retinal detachment was found at the site of inadvertent puncture.
Tubek et al.^18^	1	21	F	Poland	Black	Unknown	Inadvertent puncture in the right eye with injection of ink into the anterior chamber	Yes - anterior chamber washout followed by pars plana vitrectomy.	No	Light perception	Developed persistent ocular hypertension and significant cataract. Also had conjunctival lumps in the asymptomatic left eye similar to those in Brodie et al.^16^
Jalil et al.^19^	1	49	M	UK	Blue	Unknown	Inadvertent puncture with injection of ink into vitreous	Yes - pars plana vitrectomy with silicone oil tamponade. Later developed proliferative vitreoretinopathy, needed additional vitrectomy and long-term silicone oil tamponade.	No	Unknown	CT scan demonstrated an IOFB appearance possibly due to subretinal concentration of crystals, found to be titanium dioxide and copper containing particles.

Except for a single case of scleral tattooing using “fingerpaint,”^14^ all other cases of scleral tattooing have used commercial tattoo ink. We describe two unique cases of scleral tattoos using insulin needles and ink from a pen.

## 2 Findings

### 2.1 Case 1

A male in his 20's was brought to the emergency room with bilateral eye pain, swelling, and discharge for two to three weeks. He reported that three weeks prior while incarcerated, he drained a black ink gel pen into an insulin needle and injected the ink underneath the conjunctiva of both eyes. Initially, the patient had no symptoms, but 6–8 days after tattooing he began to experience drainage and pain from both eyes. He was initially evaluated by an outside provider and was given a short course of oral azithromycin without improvement.

**Fig. 1:**
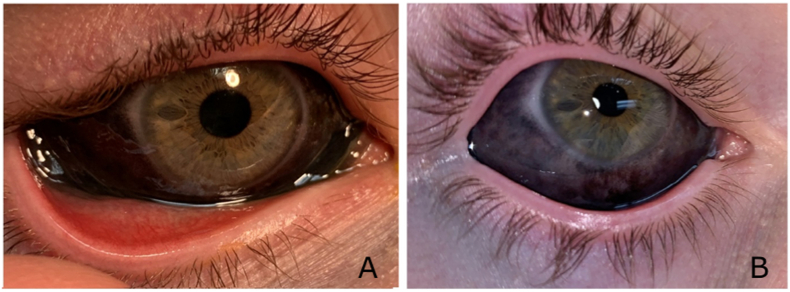
Case 1 left eye in panel A at initial presentation and panel B at 6-week follow up showing densely pigmented conjunctiva and faint hyperpigmentation of the periocular skin improving over time.

Uncorrected visual acuity on initial presentation to the ophthalmologist was 20/20 in both eyes. Intraocular pressures were 22 mmHg in the right eye and 23 mmHg in the left eye. He had mild eyelid edema in both eyes, bilateral 360-degree black conjunctival pigmentation and chemosis greater on the left side (Fig. 1). Fluorescein exam revealed small inferior focal areas of uptake corresponding to reported injection sites in both eyes which were Seidel-negative. Both corneas were clear and the anterior and posterior segment exams revealed no inflammation and were unremarkable in both eyes. A CT scan of the orbits showed formed globes and no foreign bodies in either eye. His symptoms and examination represented a chemical or allergic contact conjunctivitis.

The patient was prescribed dexamethasone/neomycin sulfate/polymyxin B sulfate ointment four times a day and moxifloxacin drops four times a day in both eyes. He returned to clinic two weeks later with improved chemosis and symptoms. The moxifloxacin drops were discontinued, and the ointment slowly tapered over the next 6 weeks. The patient was seen again 6 weeks later off all eye medications and had complete resolution of symptoms. Examination at that time included 20/20 vision in both eyes, normal intraocular pressures, and no persisting chemosis. Subconjunctival ink/pigmentation was still present, but notably reduced. Faint hyperpigmentation of the lower eyelid skin was noted in both eyes as seen in Fig. 1.

### 2.2 Case 2

A male in his 20's presented to the ophthalmology clinic with one month of itching and irritation in both eyes. He denied pain or any decrease in vision. The patient reported draining a black gel pen into an insulin needle and, with the help of another inmate, injected the ink into both conjunctivae approximately one month prior. He had been using erythromycin ointment sporadically in both eyes for approximately 3 weeks, prescribed by an outside provider soon after the tattooing.

**Fig. 2:**
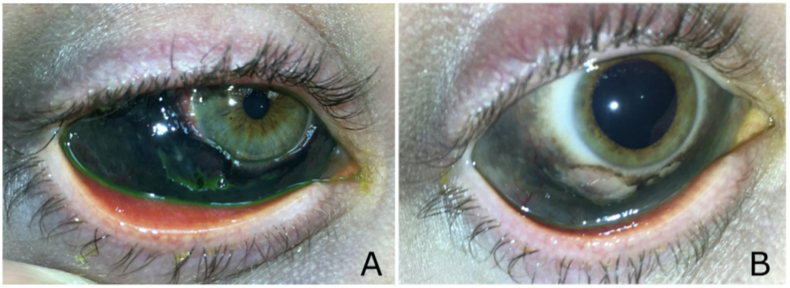
Case 2 left eye on initial presentation in panel A showing densely pigmented conjunctiva and faint hyperpigmentation to the periocular skin. The left eye after 3.5 months is shown in panel B with fading conjunctival pigmentation and periocular skin pigmentation.

Initial exam included uncorrected visual acuity of 20/30 in the right eye and 20/40 in the left. Intraocular pressures were 13 mmHg in the right eye and 14 mmHg in the left. Both eyes had mild eyelid edema with a faint discoloration of the eyelid skin. The right eye had 360° of densely pigmented conjunctiva and mild chemosis, a 2.5mm diameter conjunctival defect inferiorly, and scattered sub conjunctival hemorrhage (Fig. 2A). The left eye had 360° of densely pigmented conjunctiva and mild chemosis, a small 1.5mm diameter conjunctival defect superotemporally, and subconjunctival hemorrhage. The remaining anterior and posterior segment exams of both eyes were unremarkable and with no inflammation.

Treatment with erythromycin ointment four times a day was continued, with encouragement to improve adherence. Two weeks later, the conjunctival defects in both eyes had resolved. Three months later, the patient had developed moderate conjunctivochalasis inferiorly in both eyes. The vision at this visit improved to 20/20 in both eyes. A 2mm ring of conjunctiva around the limbus in both eyes had returned to its normal white color and the conjunctiva was overall less deeply pigmented (Fig. 2B).

## 3 Discussion

Scleral tattooing has emerged as a novel form of extreme body modification. With only 14 cases in the medical literature to date, its complications are just becoming recognized. Traditional tattoo inks, when inadvertently injected inside the anterior chamber or vitreous, can cause devastating injury to the eye and its structures including uveitis, corneal endothelial failure, secondary glaucoma, retinal detachments, infection, and loss of eye.^9^^,^^11^^,^^13^, ^14^, ^15^^,^^17^, ^18^, ^19^ From our literature review, 10/14 (71%) cases previously reported were complicated by inadvertent globe puncture, and these cases had more serious visual consequences, especially if ink was injected while inside the eye. In cases without globe puncture, less serious side effects tend to occur and are likely related to hypersensitivity of the conjunctiva to the ink itself and include irritation and conjunctival chemosis as in our cases and others, as well as episcleral nodules.^10^^,^^12^^,^^16^^,^^18^ These are likely secondary to known iron, barium, copper, or titanium dioxide pigments in tattoo ink or other associated components of the ink formulation.

The cases presented here are unique and novel in that these tattoos were administered using gel pen ink and insulin needles. This is the first report of pen ink scleral tattoos in the literature. Although tattooing within prisons in the United States and many other countries is prohibited, up to 45% of inmates receive tattoos during their incarceration.^20^ It is possible, however, that this rate is decreasing.^21^ Prison skin tattooing is often done with improvised and non-sterile materials, including sewing needles, hypodermic needles, guitar strings, and inks from pens and melted plastics. The risks of prison tattooing include infection and transmissible diseases such as Hepatitis B and C, and possibly HIV.^22^ Pen ink for skin tattooing has been described in the dermatology literature to rarely cause localized skin reactions, some specifically to components of the ink such as Solvent Blue 36 which is commonly found in blue pen ink.^23^, ^24^, ^25^ Hypersensitivity reactions can result from traditional dermal tattooing as well, and ranges from mild irritation to granulomatous inflammation requiring tattoo removal or excision.^27^ To our knowledge there is no report of pen ink or prison tattoos causing unique or increased local side effects as compared to traditional tattoo ink.

These side effects in the skin from tattoo ink or pen ink may also manifest in the conjunctival, episcleral, and scleral tissues in the presence of ink. As Tubek et al. previously noted, if these reactions do occur and are persistent even with medical treatment, complete removal of dye-containing conjunctiva would be exceedingly difficult especially in cases with permanent tattoo ink, presenting a unique challenge.^18^

Even if scleral tattooing is uneventful, and in some cases fades with time, the staining of the conjunctival tissue can make for difficult detection of important diseases such as conjunctival melanoma, scleral icterus, scleritis, or underlying scleral thinning.^18^ Additionally, uncomplicated dermal tattoos can cause delayed systemic immune reactions, such as in the well-described entity of tattoo-associated uveitis in which patients can develop bilateral uveitis and granulomatous inflammation of tattooed skin, usually occurring at least after 6 months after tattooing.^26^ Although there have already been reports of immediate anterior uveitis after scleral tattooing, there have been no reports of an uncomplicated scleral tattooing presenting with delayed tattoo-associated uveitis so it is unknown whether the proximity of the ink may increase this risk.^13^

As seen in our cases and two others, spontaneous migration of dye into the periocular soft tissues can occur.^10^, ^18^ It is difficult to know whether it is possible for the inks to similarly migrate through an intact sclera and directly affect intraocular structures.

## 4 Conclusions

We present two cases of scleral tattooing using insulin needles and pen ink. Scleral tattooing can be fraught with procedural complications and can be potentially blinding if globe penetration or infection occurs. What we can gain from these two cases is that pen ink scleral tattoos evolve quite rapidly compared to other tattoo methods, with the ability to clear the ink from the subconjunctival space. Evidenced by our cases and others, uncomplicated scleral tattooing can be treated conservatively with various topical medications, but every patient should be thoroughly evaluated with a dilated fundus exam due to risk of inadvertent globe penetration. Insight into the long-term complications of scleral tattooing remains to be seen, and even seemingly uncomplicated tattooing may have long term consequences. One of the more concerning consequences of scleral tattooing could be the masking of underlying ocular surface malignancy, though this has not yet been reported. We caution patients against the practice of scleral tattooing due to the very real danger of permanent blindness or loss of eye and advise patients to seek medical attention immediately for any adverse events.

## Patient consent

Written consent to publish this case has not been obtained. This report does not contain any personal identifying information. The Colorado Multiple Institution Review Board (COMIRB) has reviewed and approved the images and descriptions contained in this case series, COMIRB protocol #20–0887, and have deemed the information provided in the case report as unidentifiable.

## Funding

No funding or grant support was provided for this study.

## Authorship

All authors attest that they meet the current ICMJE criteria for authorship.

## Declaration of competing interest

The following authors have no financial disclosures: AR, KC, CI.
